# In Situ Printing and Functionalization of Hybrid Polymer-Ceramic Composites Using a Commercial 3D Printer and Dielectrophoresis—A Novel Conceptual Design

**DOI:** 10.3390/polym13223979

**Published:** 2021-11-17

**Authors:** Georgios Tselikos, Shahid Rasul, Pim Groen, Chunchun Li, Jibran Khaliq

**Affiliations:** 1 Novel Aerospace Materials Group, Faculty of Aerospace Engineering, Delft University of Technology, Kluyverweg 1, 2629 HS Delft, The Netherlands; gtselikos@hotmail.com; 2 Department of Mechanical and Construction Engineering, Faculty of Engineering and Environment, Northumbria University, Newcastle upon Tyne NE1 8ST, UK; shahid.rasul@northumbria.ac.uk; 3 Guangxi Key Laboratory of Optical and Electronic Materials and Devices, College of Material Science and Engineering, Guilin University of Technology, Guilin 541004, China; lichunchun2003@126.com

**Keywords:** additive manufacturing, in situ alignment, electrically assisted 3D printing, hybrid composites, 3D printing

## Abstract

Three-dimensional printing-based additive manufacturing has emerged as a new frontier in materials science, with applications in the production of functionalized polymeric-based hybrid composites for various applications. In this work, a novel conceptual design was conceived in which an AC electric field was integrated into a commercial 3D printer (-based fused filament fabrication (FFF) working principle) to in situ manufacture hybrid composites having aligned ceramic filler particles. For this work, the thermoplastic poly lactic acid (PLA) was used as a polymer matrix while 10 vol% KNLN (K_0.485_Na_0.485_Li_0.03_NbO_3_) ceramic particles were chosen as a filler material. The degree of alignment of the ceramic powders depended upon print speed, printing temperature and distance between electrodes. At 210 °C and a 1 kV/mm applied electric field, printed samples showed nearly complete alignment of ceramic particles in the PLA matrix. This research shows that incorporating electric field sources into 3D printing processes would result in in situ ceramic particle alignment while preserving the other benefits of 3D printing.

## 1. Introduction

With the global crisis surrounding the COVID-19 pandemic, additive manufacturing (AM) or 3D printing has emerged as a favorable manufacturing technique owing to its flexibility, portability, on-demand character, low cost, and use of a minimal workforce [1,2,3]. Composites have also been successfully manufactured using AM, in addition to polymers, ceramics, and metals. [4]. According to IDTechEx [5], the market for composite manufacturing using 3D printing is projected to balloon to $1.7 billion by 2030 which makes 3D printing of composites an attractive area for researchers and industries.

The materials extrusion technique for 3D printing, which is commonly referred to as fused filament fabrication (FFF), is commonly used to print hybrid polymer–ceramic particulate composites [6,7]. Polylactic acid (PLA) is a preferred choice for 3D printing of composites owing to its low melting temperature, ease of processing, low cost, and biodegradability [8,9]. For FFF processing, filaments are produced beforehand, and the process generally involves the dissolution of the polymer matrix in a solvent followed by mixing with ceramic particulates and subsequent evaporation of the solvent [10,11]. Solvent evaporation during the 3D printing process remains a challenge as it can cause warping and lengthens the processing time [12]. Hence, the extended processing time is required to match the composites manufactured by traditional methods [13]. Therefore, hybrid composite filament-processing, which uses a solvent-free manufacturing process, remains a research gap to be explored in order to minimize the environmental footprint of dying the solvents, reduce the processing time cost, and improve the properties of the printed composites.

Functional composites, such as hybrid piezoelectric composites, have also been manufactured using 3D printing and extensive work has been carried out to manufacture and embed sensors/microsystems using 3D printing [14,15,16,17,18]. Research related to additive manufactured piezoelectric materials is mainly focused on polyvinylidene fluoride (PVDF)-based materials owing to these being the most favorable piezoelectric properties for polymeric-based materials [19,20,21,22]. However, PVDF-based materials pose a challenge as PVDF is piezoelectrically active only in the β phase. As the default phase of PVDF is α, the transformation into β phase requires enhanced processing, e.g., use of organic solvents [22,23]. However, even after using enhanced processing techniques, PVDF-based composites demonstrated a low piezoelectric charge coefficient value (d_33_) value (0.1 pC/N) [24] compared to the value achieved using epoxy-based composites (in the range of 6–14 pC/N, depending upon the filler content) [25].

Three-dimensional printed hybrid piezoelectric composites face an additional challenge because of the random distribution of ceramic particles within these composites. Inducing anisotropy in these composites during manufacturing enhances piezoelectric properties compared to composites having random distribution of ceramic filler. One example of inducing the functionalization of hybrid composites is through engineering the microstructure of the composite which is composed of aligned ceramic particles within a polymer matrix [25,26]. Applying an AC electric field during dielectrophoresis (DEP) processing, for example, increases d_33_ by a factor of two and the piezoelectric voltage coefficient, (g_33_), by a factor of three as compared to those with random particle orientation [27]. The best piezoelectric properties have been reported in composites containing nearly 10 vol.% of ceramic filler in polymer matrix. At this vol.%, there exists a unique combination of low dielectric constant and d_33_ which results in enhanced g_33_ values [28,29].

There have been several reports in which piezoelectric composites have been manufactured using 3D printing, however, to the best of our knowledge, in situ alignment of ceramic particles during 3D printing of hybrid composites using an electric field has never been reported.

Therefore, in this work, to the best of our knowledge, for the first time, a conceptual design on how to use an alternating electric field to simultaneously 3D print a polylactic acid-K_0.485_Na_0.485_La_0.03_NbO_3_ (KNLN) composite with aligned KNLN particle using a solvent-free 3D printing process will be presented.

## 2. Materials and Methods

### 2.1. Materials

In this work, PLA pellets from Corbion (Gorinchem, The Netherlands) and KNLN synthesized at TU Delft (Delft, The Netherlands) using a two-step solid state calcination process [29] were used as polymer matrix and ceramic filler, respectively.

### 2.2. Method for Filament Extrusion

PLA pellets were initially crushed to form a fine powder and subsequently dry mixed with 10 vol.% of KNLN powder using zirconia balls on a rolling bench without using a solvent. As explained earlier, 10 vol.% was chosen owing to its better properties compared to other volume fractions. To extract any absorbed moisture, the mixed powders were dried overnight (minimum 12 h) at 100 °C. The filament for 3D printing was manufactured using a NEXT Regular (3devo, Utrecht, The Netherlands) filament extruder using pre-set extrusion parameters for PLA. The composite filament’s theoretical density was estimated to be 1.55 g/cc, and its actual density was determined using Archimedes’ principle.

### 2.3. Method for 3D Printing

In order to estimate the optimum printing temperature for fully dense composites, Ultimaker’s 2+ (Utrecht, The Netherlands) 3D printer was used to manufacture piezoelectric composites at temperatures ranging from 180 °C to 210 °C using a nozzle diameter of 0.6 mm.

### 2.4. Method for In Situ 3D Printing and Dielectrophoresis (DEP)

**Figure 1:**
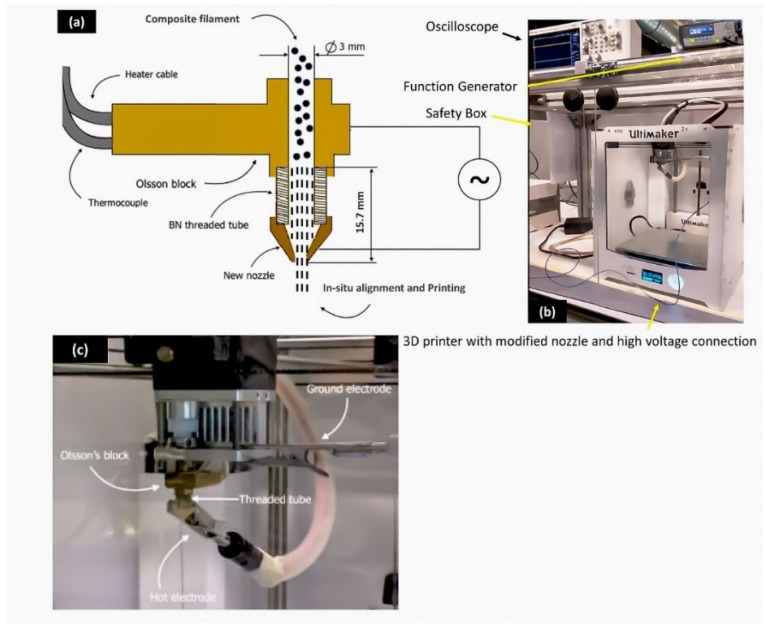
(**a**) Schematic representation of a modified Olson block for dielectrophoresis; (**b**) complete set-up of in situ printing and functionalization; (**c**) a close-up view of nozzle and electrode setup on the 3D printer.

For in-situ 3D printing and alignment of ceramic filler, the printing nozzle of the 3D printer was re-designed and modified to accommodate electric field which was then used to perform alignment using DEP processing. Three-dimensional printing nozzles were housed inside an assembly called an Olson Block that can be swapped out and has a built-in heater to melt the polymer for printing. It can be retrofitted to the entire 3D printer, making nozzle replacements simple and painless. The modified nozzle was manufactured in the workshop to apply the required electric field for particle alignment using so called ‘’top and bottom’’ electrode. The threaded portion (top part) of the nozzle was removed from the nozzle’s bottom part and new threads were made inside the bottom part in order to insert and fasten an electrically insulating threaded boron nitride tube which was crucial to insulate the bottom part of the modified nozzle from the rest of the Olson block to avoid short circuiting as they would act as top and bottom electrodes. The modified Olson block assembly was connected to an external power supply/amplifier to apply a required high voltage where the nozzle served as one electrode connected to a high-voltage cable and the Olson block as the second electrode connected to the ground cable. A schematic of the original Olson block together with the tailored 3D printer nozzle is shown in Figure 1a. Figure 1b shows the complete set up within a Perspex safety box to prevent any unwanted accidents. A close-up view of the modified nozzle together with the electrical connections is shown in Figure 1c.

### 2.5. Piezoelectric Characterisation

The composite samples containing PLA and KNLN were 3D printed in disc format and subsequently polished using sandpaper (grit size 320) to remove the top and bottom layers to enhance the roughness for better electrode adhesion. To fabricate the electrodes from 3D-printed composite discs, a Quorum Q300T sputter coater (London, UK), was used to sputtered gold on both sides of the disc. The dielectric constant (ε_r_) was calculated using the following equation.

(1) $$\varepsilon_{r}=\frac{Cd}{A\varepsilon_{o}\;}$$

where C is capacitance, measured using an LCR meter, A is the area of electrode, d is the sample thickness and ε_o_ is permittivity of vacuum which was taken as 8.854 × 10^−12^ F·m^−1^.

The composite samples were then poled for 30 min at 100 °C under a DC field of 10 kV/mm using a previously reported method [30]. A Jeol JSM-7500F scanning electron microscope (Jeol Ltd., Tokyo, Japan) was used to view the samples in cross section (SEM). A PM300 d_33_ PiezoMeter System (Piezotest, Singapore, Republic of Singapore) was used to determine the piezoelectric charge coefficient (d_33_).

## 3. Results

**Figure 2:**
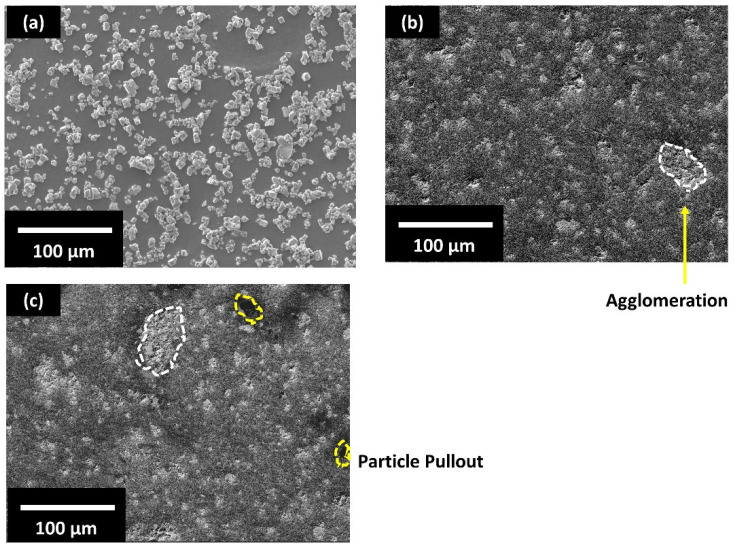
Scanning electron microscopy (SEM) image of: (**a**): KNLN (K_0.485_Na_0.485_La_0.03_NbO_3_) powder; (**b**) horizontal cross section of the filament containing polylactic acid (PLA)-10 vol.% KNLN composite; (**c**) vertical cross section of the filament of PLA-10 vol.% KNLN composite (horizontal); white circles indicate agglomeration while yellow circles indicate particle pull-out.

SEM micrographs of KNLN powder are shown in Figure 2a. Most of the KNLN particles have a size in the range of 1–4 µm with few aggregates/large particles having a particle size around 10 µm. Figure 2b,c shows the horizontal and vertical cross sections of the PLA-KNLN composite filament, respectively, prepared through the NEXT regular filament extruder. The images show that KNLN was dispersed homogeneously inside the polymeric matrix which is crucial for smooth printing of the composite. Few large aggregates, marked as white circles, were also seen which are in agreement with previous reports [29]. It is expected that during the printing of the composite, the agglomeration can be further reduced due to additional mixing within the 3D printer [31]. Particle pull-out can also be seen (marked in yellow circles) which is the result of particles being removed during grinding of the samples. Moreover, since the filament was being extruded vertically, particle alignment due to gravitational force might have taken place; however, no form of alignment was observed in the composite filament either in vertical or horizontal cross section which demonstrates that gravity has a minimum influence on the filament extrusion process when using ceramic particles with particle size in the range of a few microns.

**Figure 3:**
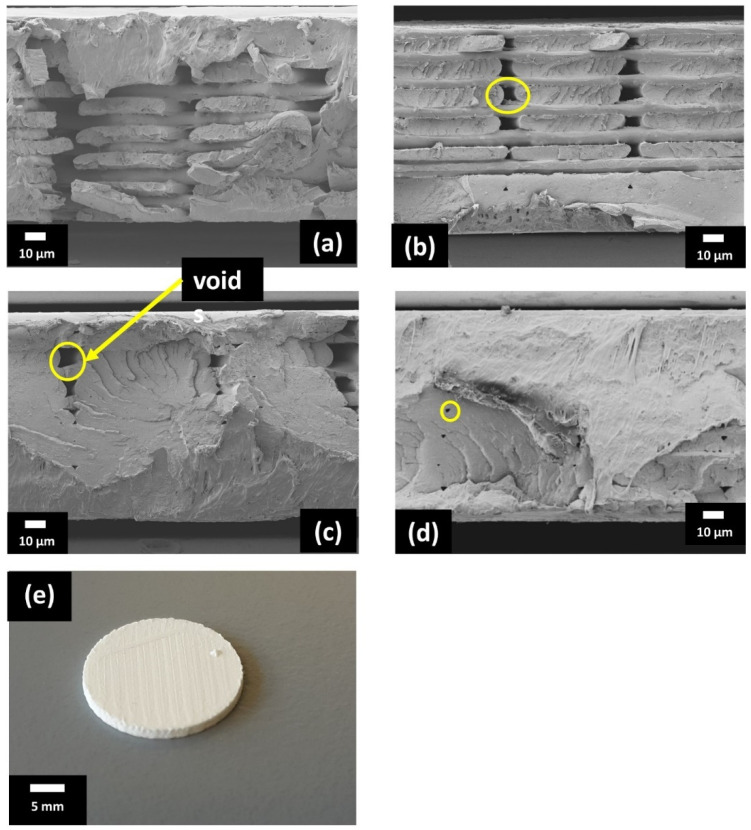
SEM images of cross-section of PLA-10 vol.% KNLN composite disc printed at: (**a**) 180 °C, (**b**) 190 °C, (**c**) 200 °C, (**d**) 210 °C, (**e**) 3D-printed composite disc.

SEM micrographs of cross-sections of 3D printed composite discs with random orientation of filler printed at a variety of temperatures (from 180 °C to 210 °C) with a nozzle diameter of 0.6 mm are shown in Figure 3a–d. The 3D print density of the composites gradually improved with increasing nozzle temperature resulting in a denser composite with fewer voids. At 180 °C, the print density of the composite was poor with a large number of voids distributed throughout the composite (marked yellow). The voids gradually decreased with increasing temperature up to 210 °C giving a nearly fully compact composite disc with a few voids as shown in Figure 3d. A relative density of 94% was found in the 3D-printed composite at 210 °C. A 3D printed composite having a random orientation of KNLN filler particles is shown in Figure 3e.

**Figure 4:**
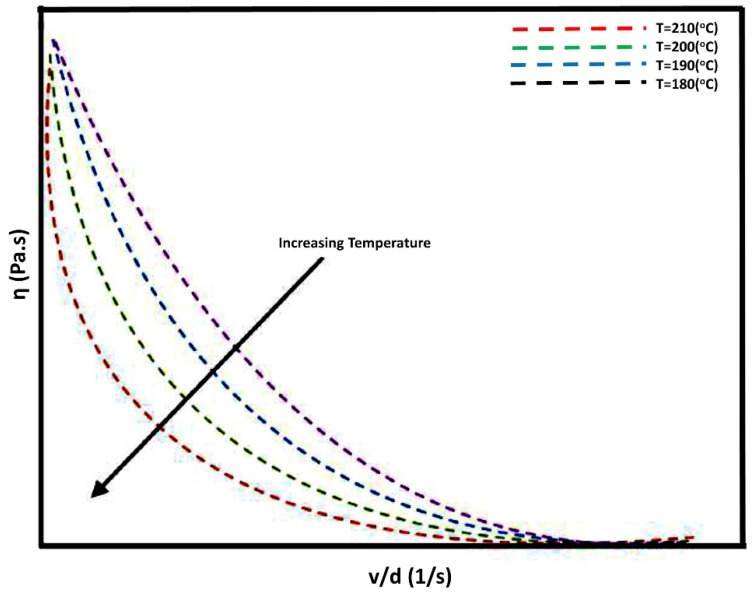
Correlation between printing speed/tube filament diameter and shear viscosity.

To develop a conceptual design for in situ 3D printing and alignment DEP method, it is important to calculate certain parameters such as distance between two electrodes which would ultimately affect the electric field reaction time of ceramic filler between the two electrodes. The time the ceramic filler is exposed to the applied electric field for alignment is referred to as reaction time which follows a linear relationship with the print speed. The reaction time should be long enough for a ceramic particle to form aligned chains inside the molten polymer. The particle’s velocity during DEP processing depends upon an equilibrium between drag force (F_drag_) and the force exerted by electric field (F_DEP_) [27]. Another parameter is the viscosity of PLA which should be low enough to overcome the drag force exerted by the molten PLA on KNLN particles during application of an electric field. The shear viscosity of PLA directly influences electric field alignment, and a higher viscosity will result in a higher drag force that acts on the particle, which in turn results in the random distribution of particles instead of particle alignment. In a dynamic system, such as 3D printing, one cannot ignore the contribution of speed of printing. The printing speed (ν) can be linked with shear viscosity of the mixture (η_c_) using

(2) $$\eta_{c}=\frac{\sigma_{c}\beta }{v}$$

where β is the tube diameter and is assumed to have a constant value of 3 mm and σ_c_ is the shear rate. Figure 4 presents the inverse relationship between printing speed and the shear viscosity at various temperatures. The curves follow the pattern of a non-Newtonian fluid that exhibits shear-thinning behaviour, which is in accordance with the behaviour of PLA [32]. Therefore, it is anticipated that the increase in temperature will reduce shear viscosity leading to lower values of F_drag_ being exerted on the particles. From the above we can conclude that the viscosity inside the in situ printing/DEP setup is influenced by both temperature and printing speed.

**Figure 5:**
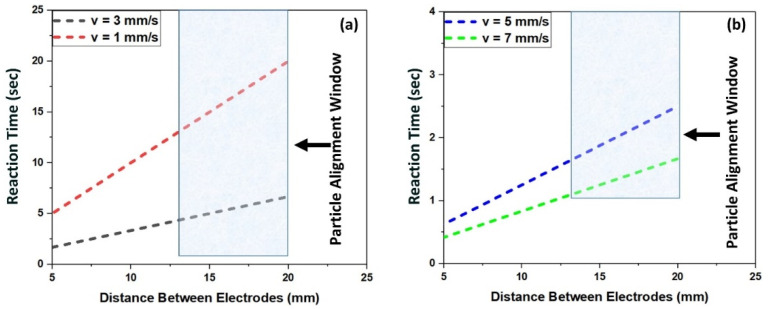
Correlation between reaction time and distance between the electrodes for printing speeds of: (**a**) 1 and 3 mm/s, and (**b**) 7 and 5 mm/s.

Within the limitations of the designed setup, a minimum length of 12.67 mm could be achieved between the electrodes when the top and bottom electrodes in the nozzle barely touch each other while a maximum length of 20 mm could be achieved when the system (electrodes) is fully extended. The reaction time can be calculated for a range of printing speeds (between 1mm/s and 10mm/s) by adjusting the length between the electrodes. Assuming PLA is already in a molten state, at a printing speed of 7 mm/s, the shortest reaction time (within the limitations of the proposed setup) that can be achieved at which alignment could be realized was calculated to be ~1–1.5 s [27], and the longest was calculated to be in the range of 12.67–20 s, for a 1mm/s printing speed as shown in Figure 5a,b. As this is a dynamic system, particles need a higher reaction time to align and form a chain-like structure. If the minimum length of top/bottom electrodes is chosen together with the minimum printing speed, a reaction time of 12.7 s could be achieved which is the maximum reaction time within the limitation of the current set-up through which alignment of particles could be realized.

**Figure 6:**
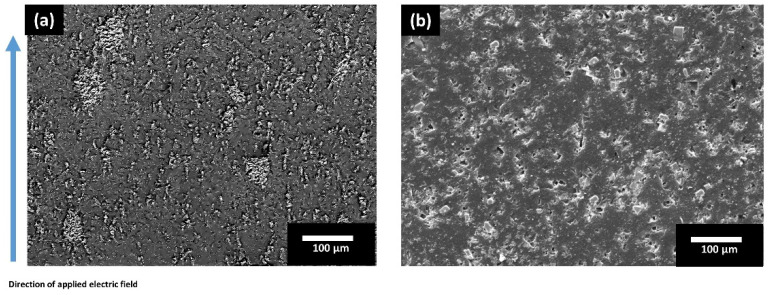
SEM micrographs of the control sample displaying the cross-section of KNLN-PLA composite: (**a**) after dielectrophoresis (DEP) process under 1 kV/mm and 1 kHz at 210 °C; (**b**) without electric field.

Another important factor for in-situ 3D printing under DEP was to estimate the optimal magnitude of applied electric field and applied frequency at the printing temperature so that the alignment of filler particles under an applied electric field could be verified. The phase angle between applied voltage and leakage current at the dielectrophoresis frequency can be used to estimate dielectrophoresis alignment efficiency. When the phase angle in the molten/uncured composite slurry is 90°, the best combination of electric field and frequency is attained, however, the phase angle can go down to ~50°. In order to estimate these parameters, a control experiment outside the 3D printer was designed which was based on an earlier report of high-temperature DEP [33]. For piezoelectric composites, DEP studies were conducted at 500 V/mm, 1 kV/mm, 1.5 kV/mm, and 2 kV/mm. For all samples, the frequency was set to 1 kHz based on the previous reports [29,30]. The level of voltage is directly affected by the distance between the two electrodes as it determines the electric field applied across the electrodes. The chosen processing temperature was kept at 210 °C in line with the initial 3D printing experiments with randomly aligned ceramic particles. Using a cold press, the pre-mixed PLA and KNLN powder were pressed into 15 mm diameter pellets and heated to 210 °C under an electric field ranging from 500 V/mm to 2 kV/mm and 1 kHz. An electric field of 1 kV/mm was found to be optimal for PLA-KNLN composites as no alignment was observed for 500 V/mm and at an electric field 2 kV/mm, the polymer showed significant overflow due to overheating which was caused by dielectric loss heating [34] resulting in samples unsuitable for use. Figure 6a displays SEM images of the cross-sections of PLA-KNLN composites with 10 vol.% KNLN manufactured using a high temperature DEP method under an electric field of 1 kV/mm together with a frequency of 1 kHz at 210 °C. Staggered distribution of particles can be observed inside the microstructure with chain-like formation and no sedimentation was observed. This demonstrated that particles alignment can be achieved at temperatures of 210 °C and higher for in situ 3D printing and DEP. For comparison, a sample without electric field was also manufactured and shown in Figure 6b. It can be clearly seen that the particles have random orientation compared to the sample manufactured using an electric field. According to the linear relationship between the time for which particles were exposed to an electric field and applied voltage, an electric field of 1 kV/mm requires an application of 12.67 kV when the device is minimally extended and can go up to 20 kV when the system is fully extended with 20 mm between two electrodes.

**Figure 7:**
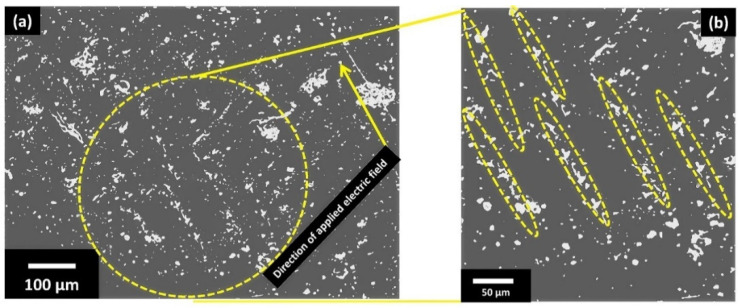
(**a**) Scanning electron microscope image of cross section of in-situ 3D printed and DEP functionalized PLA-10 vol% KNLN composite under 1 kV/mm at 210 °C; (**b**) magnified view.

The theoretical estimation and experimental work presented above provided a concrete set of parameters for the development of 3D printing and DEP setup for in situ alignment/functionalization of KNLN particles in the PLA matrix. The distance between the top and bottom electrode was kept at 15.7 mm at a printing temperature of 210 °C to allow a reaction time of ~15 s. A frequency of 1 kHz with an electric field of 1 kV/mm (voltage of 15.7 kV) was applied which is also consistent with the previous research on polymer based piezoelectric composites [25,30]. As soon as the composite filament melted inside the nozzle, the electric field aligned the particles in polymer matrix before it solidified on exiting the nozzle. The non-AC electric field polarized the dielectric KNLN particles which then experienced a force along the arbitrary electric field lines. As the AC electric field was non-uniform in nature, the newly created dipoles would be experiencing an unequal electric field owing to the size difference [35]. As a result, the dipole experiencing the dominant electric field started to move towards the neighboring electrode or the particle of opposite polarity hence creating a chain like structure. The cross-section of the composite (along the electric field direction) was examined using the SEM to verify the alignment of the ceramic particle. A non-uniform chain-like formation of the ceramic particles was observed along the direction of the electric field as indicated in Figure 7a which clearly indicated the alignment and proved the concept of the engineering design. The non-uniform alignment of particles could be attributed to the enhanced temperature during printing because of electric field resulting in reduced viscosity of PLA [36]. Figure 7b zooms into a specific area that clearly demonstrates the alignment of particles.

One of the performance criteria for a fully dense active piezoelectric composite with random orientations of the filler material is to have a dielectric constant comparable to that calculated using Yamada’s model for biphasic/hybrid composites. Dielectric constant depends directly on the density of the composite as the presence of void (air) decreases the dielectric constant (ԑ_r_) due to the lower dielectric constant of the air [37]. According to Yamada’s model, ԑ_r_ for randomly oriented composites can be calculated by [38]:

(3) $$\varepsilon_{r}=\varepsilon_{p}\left(1+\frac{n\phi \left(\varepsilon_{C}-\varepsilon_{P}\right)}{n\varepsilon_{p}+\left(\varepsilon_{C}-\varepsilon_{p}\right)\left(1-\phi \right)}\right)$$

where ԑ_p_ is the polymer’s dielectric constant, n is the ceramic particles’ aspect ratio, ϕ is the ceramic filler’s volume fraction, and ԑ_c_ is the ceramic filler’s dielectric constant. The ԑ_r_ value was calculated using ԑ_p_ = 3.14, ϕ = 10, n = 5.6 and ԑ_c_ = 400 which returned ԑ_r_ value of 5, in close agreement with the experimental value of 5.3 [39]. Furthermore, the poled composite’s piezoelectric charge coefficient was measured using a Berlin court meter, yielding a value of 1.35 ± 0.21 pC/N which is equivalent to state-of-the-art epoxy-PZT composites at identical volume fractions [27]. Based on the ε_r_ and d_33_ values, piezoelectric voltage coefficient g_33_ was calculated using equation g_33_ = d_33_/ε_r_ε_o_ and a g_33_ value of 28.6 ± 0.9 mVm/N was obtained for randomly oriented 3D printed which is comparable to the values reported for bulk ceramics [25].

**Figure 8:**
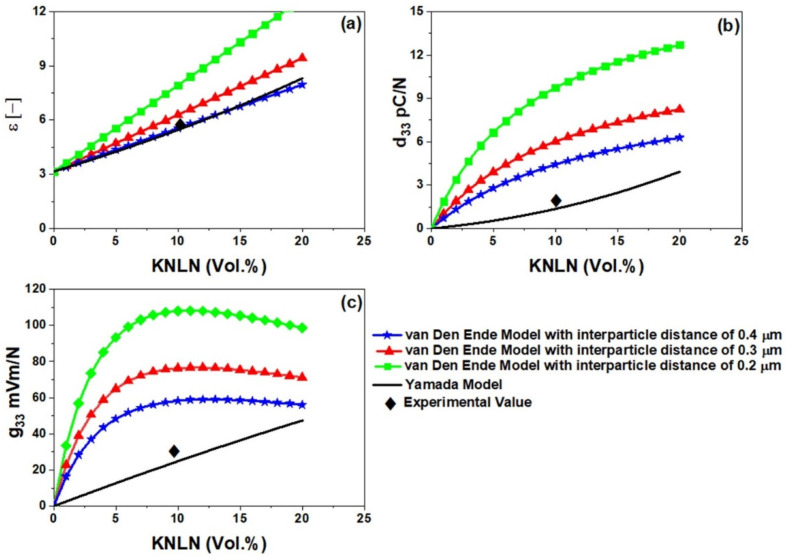
Calculated values of piezoelectric properties using van Den Ende (for composites with aligned particles) and Yamada (for composite with random orientation of particles) model: (**a**) dielectric constant (ε_r_); (**b**) piezoelectric charge coefficient (d_33_); and (**c**) piezoelectric voltage sensitivity (g_33_).

In order to calculate the estimated piezoelectric properties for in situ 3D printed and DEP aligned composite, van den ende model [27] and Bowen model [40] were used to calculate d_33_ and ε_r_, respectively, through the following two equations:

(4) $$d_{33}=\frac{{\left(1+R\right)}^{2}\varepsilon_{p}\phi Y_{33_{c}}d_{33_{c}}}{\left(\varepsilon_{c}+R\varepsilon_{p}\right)\left[\left(1+R\phi \right)Y\varepsilon \varepsilon_{p}+\left(1-\phi \right){\mathrm{RY}}_{p}\right]}$$

(5) $$\varepsilon_{r}=\phi \;(\frac{R\varepsilon_{p}\varepsilon_{c}}{\left(\varepsilon_{c}+R\varepsilon_{p}\right)})+\left(1-\phi \right)\varepsilon_{p}$$

where R is the average particle size to inter-particle distance ratio, Y_p_ and Y_33c_ are the polymer and ceramic elastic moduli, and d_33c_ is the ceramic’s piezoelectric charge coefficient. The van den ende model has previously been shown to be able to predict dielectric values for DEP-aligned composites. As 3D printing is a dynamic process, the interparticle distance between ceramic fillers can change and will not be a fixed entity. To estimate the dielectric properties of in situ 3D-printed and DEP-aligned composites, an interparticle distance of 0.3 µm, 0.4 µm, and 0.5 µm based on SEM image analysis and earlier reports were considered [29]. Figure 8a–c shows a comparison of dielectric constant, d_33_, and g_33_ values at different interparticle distances. As can be seen, decreasing interparticle distance has an increasing effect on piezoelectric properties. The interparticle distance would obviously decrease with increasing filler content, however, at the cost of a higher dielectric constant. Most importantly, DEP aligning has a more pronounced effect on d_33_ rather than dielectric constant which in turn produces higher g_33_ values as can be seen in Figure 8c. In situ aligned 3D printed under DEP composites are estimated to have higher values compared to the 3D printed composites with random orientations, however, to verify these estimated values, a further enhancement and optimization of in situ 3D printing and DEP setup are required.

## 4. Conclusions

In summary, a novel engineering concept of in situ alignment of ceramic particles in a polymer matrix for a 3D-printed hybrid composite disc sample using an AC electric field was demonstrated, prototyped, and incorporated into a commercial 3D printer. The applied electric field, printing speed and printing temperature were found to affect the degree of alignment of the piezoelectric ceramic powders. Dielectrophoresis was used in conjunction with a commercial 3D printer to improve the rheological and printing properties of a hybrid polymer–ceramic composite, demonstrating that ceramic particles can be aligned during the printing process of a polymeric matrix at 210 °C by applying an electric field of 1 kV/mm, at a frequency of 1 kHz. Anisotropic hybrid composites with improved unidirectional properties can be produced at a commercial scale using the in situ 3D printing and DEP setup built in this research. The anisotropic properties of the in situ 3D printed hybrid composites along with their ease of manufacturing can find a range of industrial applications, such as microelectronics, energy storage/harvesting systems, and biomedical applications.
